# Developmental and genetic effects on behavioral and life‐history traits in a field cricket

**DOI:** 10.1002/ece3.4975

**Published:** 2019-02-23

**Authors:** Tina W. Wey, Denis Réale, Clint D. Kelly

**Affiliations:** ^1^ Université du Québec à Montréal Montréal Québec Canada; ^2^ Université de Sherbrooke Sherbrooke Québec Canada

**Keywords:** behavioral traits, developmental plasticity, *Gryllus firmus*, heritability, population density, quantitative genetics

## Abstract

A fundamental goal of evolutionary ecology is to identify the sources underlying trait variation on which selection can act. Phenotypic variation will be determined by both genetic and environmental factors, and adaptive phenotypic plasticity is expected when organisms can adjust their phenotypes to match environmental cues. Much recent research interest has focused on the relative importance of environmental and genetic factors on the expression of behavioral traits, in particular, and how they compare with morphological and life‐history traits. Little research to date examines the effect of development on the expression of heritable variation in behavioral traits, such as boldness and activity. We tested for genotype, environment, and genotype‐by‐environment differences in body mass, development time, boldness, and activity, using developmental density treatments combined with a quantitative genetic design in the sand field cricket (*Gryllus firmus*). Similar to results from previous work, animals reared at high densities were generally smaller and took longer to mature, and body mass and development time were moderately heritable. In contrast, neither boldness nor activity responded to density treatments, and they were not heritable. The only trait that showed significant genotype‐by‐environment differences was development time. It is possible that adaptive behavioral plasticity is not evident in this species because of the highly variable social environments it naturally experiences. Our results illustrate the importance of validating the assumption that behavioral phenotype reflects genetic patterns and suggest questions about the role of environmental instability in trait variation and heritability.

## 1 INTRODUCTION

A fundamental goal of ecological and evolutionary studies is to identify the causes of underlying interindividual trait variation. Significant areas of study include identifying the relative importance of genetic and environmental influences on the expression of phenotypes (Falconer & Mackay, 1996; Lynch & Walsh, 1998; Roff, 1997), quantifying the extent to which traits respond to developmental cues (Pigliucci, 2005; West‐Eberhard, 2003), and measuring the link between fitness and genetic variation (Mousseau & Roff, 1987; Price & Schluter, 1991; Stirling, Réale, & Roff, 2002). Traditional traits of interest in eco‐evolutionary studies were typically morphological or life‐history traits; however, considerable attention is currently being paid to questions regarding within‐population variation in behavioral traits (Dingemanse, Kazem, Réale, & Wright, 2010; Réale, Reader, Sol, McDougall, & Dingemanse, 2007; Sih, Bell, Johnson, & Ziemba, 2004; Wolf & Weissing, 2012) Existing work on quantitative genetics of behavior suggests that behavioral traits are, on average, as heritable as life‐history traits and are subject to varying degrees of selection (Boake, 1994; Mousseau & Roff, 1987; Stirling et al., 2002). Earlier research tended to focus first on mating and sexually selected behaviors (Boake, 1994), but more studies have recently tested for heritability of behaviors, such as exploration, boldness, and activity, that can systematically lead to differences in how individuals interact with their environment (Ariyomo, Carter, & Watt, 2013; Bize, Diaz, & Lindström, 2012; Brommer & Kluen, 2012; Dingemanse, Both, Drent, Oers, & Noordwijk, 2002; Niemelä, Dingemanse, Alioravainen, Vainikka, & Kortet, 2013; van Oers, Drent, Goede, & Noordwijk, 2004; van Oers & Sinn, 2011; Patrick, Charmantier, & Weimerskirch, 2013; Réale, Gallant, Leblanc, & Festa‐Bianchet, 2000; Sinn, Apiolaza, & Moltschaniwskyj, 2006; Winney et al., 2018). Heritability estimates from these studies have been variable, with behaviors not uncommonly having low values or confidence intervals overlapping zero. Thus, it remains important to test the underlying assumption of many studies that behavioral variation represents underlying genetic variation and to look for patterns in why heritability varies between different types of behavior or under different circumstances (van Oers & Sinn, 2011; Winney et al., 2018). Moreover, developmental experience can affect the heritability of adult behaviors (Dingemanse et al., 2009), but empirical tests of this are less common.

Phenotypic plasticity is the ability of one genotype to produce different phenotypes under different environmental conditions, and the combination of genetic architecture and environmental cues will affect the expression of plasticity (Pigliucci, 2005; West‐Eberhard, 2003). Behaviors will typically be more labile in expression than other types of traits, and thus, behavioral plasticity is predicted to be a particularly prevalent (Brommer, 2013; West‐Eberhard, 1989, 2003; Wolf & Weissing, 2012). However, many important questions remain about how developmental experiences affect adult behavioral phenotypes, including whether different genotypes respond differently to developmental cues (Stamps & Groothuis, 2010a, 2010b). Environmentally cued plasticity might be adaptive in variable environments. If cues during development are likely to predict adult conditions, genotypes that can respond to developmental cues might be better suited to adult environments. Social cues during development are postulated to be especially important for adaptively adjusting adult behavioral phenotypes to variable social and competitive environments (Kasumovic, Bruce, Herberstein, & Andrade, 2009; Kasumovic & Brooks, 2011; Sachser, Kaiser, & Hennessy, 2013), and gene by social environment interactions are expected to be particularly complex. On the other hand, we may fail to see environmentally cued plasticity if developmental cues are unlikely to predict adult conditions. This might occur when environmental conditions are likely to be so changeable on short timescales (within an organism's lifespan) or if organisms are able to disperse away from natal conditions (Clobert, Baguette, Benton, Bullock, & Ducatez, 2012).

Social density can have broad‐ranging effects in many insects, including eliciting changes in morphology and behavior (Applebaum & Heifetz, 1999). Often, higher density results in faster development and smaller size, as well as more active individuals, although with exceptions (reviewed in Applebaum & Heifetz, 1999). Both larval and adult density can affect behaviors such as boldness and activity (Müller, Küll, & Müller, 2016; but see also Niemelä, Vainikka, Lahdenperä, & Kortet, 2012b). In the current study, we examined the relative influence of genotype and environment on adult expression and heritability of behavioral and life‐history traits in sand field crickets (*Gryllus firmus*). *Gryllus firmus* naturally inhabits ephemeral habitats, and individuals can experience high variation in physical and social environments. Individual females will lay both fast‐developing and diapause eggs, likely as an adaptation for this variable environment (Walker, 1980). Multiple morphological and life‐history traits in this species respond to developmental density and are heritable to varying degrees (Crnokrak & Roff, 1995, 1998; Fairbairn & Roff, 1990; Roff, 1990; Roff & Gélinas, 2003; Stirling, Fairbairn, Jensen, & Roff, 2001). Some behaviors are integrated with morphology or reproductive strategies (Crnokrak & Roff, 1995; Fairbairn & Roff, 1990), but, in general, behavioral traits are less well explored in this system.

Here, we use a quantitative genetics approach with rearing density manipulations to examine the joint effects of genetics and developmental environment on the adult expression of behaviors (activity and boldness/risk‐taking), as well as morphological and life‐history traits. Boldness has been shown to be repeatable in *Gryllus integer* (Hedrick, 2000; Niemelä, Vainikka, Hedrick, & Kortet, 2012a), and boldness often correlates with activity across species (Sih et al., 2004). We test the “phenotypic gambit” of assuming that phenotypic variation reflects genetic patterns, which can be particularly tenuous for complex and plastic traits such as behaviors (Brommer & Kluen, 2012; van Oers & Sinn, 2011). Moreover, while behavioral traits show moderate heritability (~0.3) on average (Stirling et al., 2002), genetic patterns might be less predictive in a species that encounters unpredictable conditions and shows a high degree of plasticity in other traits. We tested for environmentally cue plasticity in behavior, specifically if differences in activity and boldness were induced by differences in rearing density. If so, we expected individuals raised at high densities to be bolder and more active in response (based on previous research, Applebaum & Heifetz, 1999; Niemelä, Vainikka, Lahdenperä et al., 2012b). Finally, we tested for genotype differences in phenotypic response to developmental environment, which might indicate the potential for different behavioral or life‐history strategies.

## 2 MATERIALS AND METHODS

### 2.1 Animal rearing and breeding design

We acquired *G. firmus* eggs from a laboratory stock at MacEwan University (AB, Canada), which was originally established from 35 wild‐caught adults (13 males and 22 females) collected near Gainesville, FL, in the fall of 2010 and maintained in large numbers since. The eggs used to start the current experimental colony were drawn from a large colony bin (~200 individuals of an approximately even sex ratio) and shipped to UQÀM in the fall of 2015. We maintained the current UQÀM experimental colony at a constant temperature (28°C) and humidity (60%) with 12‐hr day/night light schedule. We maintained colony animals in 70‐L mixed‐sex bins of about 50 adult individuals, provided with cotton‐plugged water vials and ad libitum Iams™ Proactive Health™ adult original cat food. Each bin had stacked cardboard egg cartons to provide refuge and wire mesh lids to provide ventilation. Minimum generation times from egg to final molt (eclosion) of offspring were less than 60 days, and the long‐winged morph was present at >90%, suggesting “optimal” growing conditions (Roff, 1990).

Beginning in the late spring of 2016, we used crickets from the second generation of the UQÀM colony (i.e., the offspring of the individuals that hatched from the eggs shipped from MacEwan) as the parents in a nested maternal half‐sib, paternal full‐sib experimental design. We randomly removed a subset of crickets from colony bins just before eclosion to insure that they had no previous mating experience and placed them individually in 250‐ml transparent square plastic containers with ad libitum food and water and a piece of cardboard egg carton for refuge. At sexual maturity (at least 7 days after eclosion), we formed mating groups with one male and three females in a 750‐ml plastic container with ad libitum food and water and several pieces of egg carton as refuge. For logistical reasons, we could not create and manipulate all the families at the same time, so we created families in seven temporal blocks, each of them composed of a median of three sires (range 2–6 in the final dataset). We kept mating groups together for 5 days before all individuals were separated and weighed. We subsequently placed females individually into containers of the same size and set‐up, provided with a small cup of damp vermiculite for oviposition. We removed females from the oviposition containers after 5 days. We kept the vermiculite in the same container and moistened until hatchlings began appearing about 12 days after laying. We removed the vermiculite after at least about 50 hatchlings had emerged, usually about 3 days. While this restricts experimental animals to earlier hatchlings, we preferred to reduce the amount of variation from this source. Hatchlings remained in the original container for 7 days, provided with powdered cat food, water, and egg cartons.

We initially created mating groups with 30 sires and 90 dams. After excluding unsuccessful females that produced no offspring and mating groups with questionable conditions (e.g., the sire died early in the mating period), we placed hatchlings from 24 sires and 68 dams into experimental density treatments.

### 2.2 Experimental treatments

After 7 days, we randomly assigned hatchlings of each maternal family to one of two density treatments. High‐density treatments consisted of groups that were started with 10 siblings in a 750‐ml transparent plastic container, while low‐density treatments consisted of one individual in a 250‐ml transparent rectangular plastic container. We note that our treatments thus necessarily represent a combination of differences in density and container size/space, so differences trait expression might be attributable to both. However, our goal was to examine the effect of social interactions on development, specifically behavioral and physiological anticipation of potential future competition, rather than to affect resource limitation or environmental stress, and small containers would have been very crowded for 10 individuals with food, water, and refuge. All animals had ad libitum food and water and ample refuge, and conditions in the high‐density treatment were relatively uncrowded until animals neared adult size. We cleaned the containers and changed food and water once per week or more often as needed throughout development.

For each dam, we created 10 low‐density and two high‐density groups (i.e., 10 offspring in low density and 20 offspring in high density), with the initial goal of testing six offspring (three males, three females) from each treatment for each dam (i.e., 12 offspring per dam and 36 offspring per sire). Some mortality during rearing and variation in sex ratio of the selected nymphs resulted in some variation in the exact numbers and sexes of experimental animals from each family. Although some high‐density groups ended with fewer than 10 individuals, we expected that these still effectively represented increased social experience, so all high‐density groups were considered valid unless they dropped to five individuals or fewer. If the combined number of offspring in high‐density treatments for a maternal family (i.e., across both original high‐density groups for a dam) fell to fewer than 10 individuals, we combined the two high‐density groups to create one group to maintain the difference in social experience between low‐ and high‐density treatments. Animals in both treatments experienced the same overall level of disturbance during regular feeding and cleaning. We moved rearing containers for all offspring haphazardly and periodically around the rearing chamber to try and minimize microhabitat variation.

Upon eclosion (the final molt to adulthood), we weighed crickets to the nearest 0.0001 g using a Sartorius (Göttingen, Germany) analytical balance and transferred them to a new 250‐ml container with food, water, and a cardboard refuge. We kept experimental animals individually for at least 7 days (to insure sexual maturity) before behavioral testing. We weighed experimental animals a second time on the day of behavior testing (described below). While adult mass is not a fixed measure of size, we used it as a simple morphological measure known to be heritable (Rantala & Roff, 2006). Our focus in this study was on behavioral traits, and we used mass to confirm expected effects of density and variance components on morphology.

### 2.3 Behavioral testing

We measured two behaviors of experimental animals—activity in an open field and latency to emerge from a refuge—using Ethovision® XT video tracking software (Noldus, Spink, & Tegelenbosch, 2001) and PhenoTyper® observation arenas (30 × 30 × 35 cm), outfitted with a built‐in infrared camera for overhead behavioral recording. We accounted for variation in the exact day of testing for experimental animals (between 7 and 14 days after eclosion) in statistical models (see Statistical analysis), but the effect was never significant.

On the night before testing, we moved test animals from the rearing chamber to the testing laboratory and placed in an incubator (28°C). Behavioral testing took place between 9:00 and 16:00 hr, during the animals' nocturnal period, in a dimmed laboratory with ambient white noise. Each animal first received an open‐field test to assess its level of activity in a novel environment. We transferred the animal from its home container to a 250‐ml plastic container and placed it in the center of an arena, with the container and opaque lid over the animal. After 2 min of acclimation, we removed the container and lid, replaced the arena cover, and started behavioral tracking (Ethovision® XT). We tracked the movement of each animal for 5 min.

After the open‐field test, we tested each animal's boldness (response to potential risk) in a manner similar to tests in related *Gryllus* cricket species (e.g., Hedrick, 2000; Niemelä, Vainikka, Hedrick et al., 2012a). We tested boldness in the same arena as the open‐field test by placing the focal animal into an opaque cylindrical plastic tube, covered with a lid and placed upright in a corner of the arena. After 2 min of acclimation, we removed the lid and gently laid the tube down on the arena floor. We then covered the arena and recorded behavior for up to 30 min. We scored videos later for the time (in seconds) required for the focal animal to fully emerge from the tube. Animals that did not emerge within 30 min (3.3% of all crickets tested) received a latency time of 1800s (i.e., 30 min), and excluding these animals did not change the interpretation of estimated effects (see also Statistical analysis). So that boldness would correlate positively with willingness to exit the tube, we subtracted each individual's (log‐transformed) latency score from the maximum value to get their boldness score. After the boldness trial, we weighed each focal animal to the nearest 0.0001 g and placed back in its home container.

We used up to four Phenotyper® units at a time; thus, most animals were tested in groups of four, where test animals were sequentially placed into arenas and sequentially manipulated with a preset amount of time between each. While there could have been slight variations among arenas, preliminary analyses suggested that there were no apparent arena or order effects on behavioral measures, so we do not consider these variables in further analyses. We distributed testing of siblings over multiple days and over different groups of four when same‐day testing was unavoidable so that all days contained multiple families and all families were tested over multiple days.

### 2.4 Statistical analysis

We fit univariate linear mixed models (LMMs) to examine the effects of family, density treatment, and sex on multiple traits: body mass at eclosion (to nearest 0.0001 g), body mass at sexual maturity (on the day of behavioral testing; to nearest 0.0001 g), development time (days from hatching to eclosion, log‐transformed), total activity in the entire 5 min. of the open field (distance in cm, square‐root transformed), activity‐per‐minute of the open field (distance in cm, natural log‐transformed), and boldness. We confirmed that all estimated effects (both fixed and random) were similar for development time and latency to emerge whether these variables were analyzed with survival models, but we report results from LMMs to allow calculations on variance components. All estimated effects for boldness were also similar if we excluded individuals that did not emerge from the analysis. We included density treatment (high or low), sex, and their two‐way interaction as fixed effects in all models. The models of behavior (boldness and activity) also included a fixed effect of days after eclosion (to account for slight differences in maturity on day of testing). The model for activity‐per‐minute additionally included fixed effects of time (minute of the trial, 1–5; standardized to mean = 0 and standard deviation = 1) and a quadratic term for time (minute^2^). We did not include the effect of wing morphology in statistical models because the great majority (91%) of test animals were long‐winged morphs.

We ran a first set of models that included random intercepts for dam nested within sire and sires nested in mating block (to identify mating groups that were formed at the same time). Models of behavioral variables (boldness, total activity, and activity‐per‐minute) further included a random intercept for day of testing. We tested for genotype‐by‐environment (G × E) interactions using a reaction norm approach, where we included random slopes for density treatment within sire with correlated intercept and slope, with high density as the intercept. We interpreted significant effects of random slopes as evidence for differences in genotype response to density. When random slopes were significant, we provide variance components for the two treatments separately (obtained from models fitted to the subsets of data on high‐ and low‐density animals separately, excluding fixed effects of density). Otherwise, we present variance components from the full dataset in the main text and provide treatment‐specific variance components in Supporting Information Tables S1 and S2.

We tested for significance (*p* < 0.05) of random effects using log‐likelihood ratio tests (LRTs) of nested models that differed only in the random effect of interest (with 1 degree or 2 degrees of freedom for intercepts and slopes, respectively). Note that LRTs represent conservative null hypothesis tests in this case, where variances are bounded at 0, and *p*‐values can be up to twice as large as they should be (Pinheiro & Bates, 2000). As this did not affect interpretation of our main results, we present unmodified *p*‐values from LRTs. We initially also tested for mother × environment (M × E) interactions by including random slopes for density within mother. However, these were not significant for any trait (all *p* > 0.15), except for maturation rate, where M × E was confounded with sire × environment (S × E), as a model with both terms did not converge. When S × E and M × E were considered in separate models of maturation rate, the model with S × E had higher likelihood and lower information criteria values, so we considered this the better model. Thus, in effect, we do not consider M × E interactions further, and GxE interactions in this study represent S × E interactions.

Additionally, for the model of activity‐per‐minute, for which we had five 1‐min observations per individual, we fit three alternate models of random effects: (a) random intercepts for individual, sire, dam, block, and test day; (b) random slopes for time within individual, with correlated intercept and slope, plus random intercepts for sire, dam, block, and test day; and (c) random slopes for time within sire, with correlated intercept and slope, plus random intercepts for individual, dam, block, and test day. Model 1 assumes no G × E or individual‐by‐time (I × T) effects on change in activity. Model 2 assumes that individuals differ in their change in activity, but that there is no G × E effect (i.e., differences are among individuals, not families). Model 3 assumes that there is a G × E effect that explains the I × T effect found in model 2. We tested models 2 and 3 against model 1 using LRTs and took the one with the greatest likelihood (and lower AIC) with *p* < 0.05 as the best model. Model 2 explained the most variance (greatest log‐likelihood and lowest AIC, *p* < 0.05); therefore, we present results from this model.

For the primary set of models, we estimated narrow‐sense heritability (*h* ^2^) as *V*_A_/(*V*_A_ + *V*_M_ + *V*_block _+ *V*_R_), where *V*_A_ is the additive genetic variance, *V*_M_ is the variance attributable to the mother above and beyond the variance among sires (*V*_S_), *V*_block_ is the variance due to difference in mating periods, and *V*_R_ is residual variance. In the nested half‐sib, full‐sib design, *V*_S_ is approximately *V*_A_/4, thus, we used *V*_A_ = 4 × *V*_S_ in our calculation of *h* ^2^. We also estimated maternal effects (*m* ^2^) as the ratio *V*_M_/(*V*_A_ + *V*_M_ + *V*_block_ + *V*_R_).

The above set of models included temporal block as a random effect. However, we could not randomize families among blocks with our experimental design, as blocks necessarily represented separate periods during which we selected some individuals to create families and each family is nested in a block. In this situation, variance among blocks may take part of the variance among sires or dams, as blocks may be composed of different genotypes due to uncontrolled factors (e.g., by chance related to the small number of parents selected in each block or for phenological reasons). Therefore, including block in the model may lead to underestimation of both *V*_A_ and *V*_M_. We thus ran a second set of models on the same dependent variables that differed only in excluding the random intercept for block effect. For this second set of models, we estimated narrow‐sense heritability (*h* ^2^) as *V*_A_/(*V*_A_ + *V*_M_ + *V*_R_). We assumed that if block really has an effect on the variable, independent of sires and dams, we should observe an increase in both *V*_A_ and *V*_M_ in models without temporal block. On the other hand, changes in only *V*_A_ or *V*_M_ as a result of removing blocks from the model would suggest that variance among blocks actually represents part of that variance component. We present results for variance components calculated from this second set of models in Supporting Information Tables S1 and S2 and discuss the implications of model differences.

We excluded any families that experienced irregular rearing conditions from these statistical analyses, resulting in 679 offspring (338 high density, 341 low density; 325 female, and 354 male) from 23 sires and 59 dams (specific sample sizes varied by analysis due to missing data points). We conducted all statistical analyses in the R statistical environmental (R Development Core Team, 2014). We implemented mixed models with the package “lme4” (Bates, Maechler, Bolker, & Walker, 2015) and survival analyses with the package “coxme” (Therneau, 2015). We used the package “boot” (Canty & Ripley, 2017; Davison & Hinkley, 1997) to obtain 95% confidence intervals and p‐values for fixed effects using 10 000 bootstrap replicates with replacement.

## 3 RESULTS

### 3.1 Variance components and heritability

Variance components and heritability estimates from models with block effect are presented in Table 1. (See Table S1 for variance components and heritability estimates from models without block effect and Table S2 for treatment‐specific components.) Mass at eclosion showed significant *V*_S_, whether effect of block was included or not, explaining 7.3% or 20.8% of phenotypic variance (*V*_P_) and with *h^2^* of 0.24 or 0.51, respectively. There was no significant *V*_M_ in either set of models, explaining <0.1% *V*_P_ and with *m^2^* <0.01 in both cases. Mass at sexual maturity only had a significant *V*_S_ when block was not included in the model, explaining 16.9% of *V*_P_ and with *h^2^* = 0.45, compared with 3.0% of *V*_P_ and *h^2^* = 0.11 when block was included in the model. There was significant *V*_M_ in both sets of models, explaining 3.8% or 3.9% of *V*_P_ and with *m^2^* = 0.04 or 0.03 in models with and without block, respectively.

**Table 1:** Variance components and narrow‐sense heritability estimates for measured traits

Variance components	Predictor	LCI	UCI	*χ* ^2^	*p*‐value
Mass, eclosion					
*V*_S_	**1,299**	209	2,708	**10.76**	**0.001**
*V*_M_	<0.01	0	670	<0.01	>0.999
*V*_block_	**2,096**	<0.01	5,984	**9.24**	**0.002**
*V*_R_	14,439	12,816	15,958		
*h^2^*	0.24				
*m^2^*	<0.01				
Mass, sexual maturity					
*V*_S_	529	0.00	1,608	1.73	0.188
*V*_M_	**689**	<0.01	1,637	**3.90**	**0.048**
*V*_block_	**2,298**	49.5	6,221	**12.25**	**<0.001**
*V*_R_	14,422	12,847	16,069		
*h^2^*	0.11				
*m^2^*	0.04				
Development time					
High density					
*V*_S_	0.0005	0	0.0022	0.35	0.556
*V*_M_	**0.0026**	0.0011	0.0043	**27.25**	**<0.001**
*V*_block_	**0.0039**	0.0002	0.011	**11.58**	**<0.001**
*V*_R_	0.0062	0.0052	0.0073		
*h^2^*	0.13				
*m^2^*	0.18				
Low density					
*V*_S_	0.0010	0	0.0040	0.08	0.783
*V*_M_	**0.0036**	0.0010	0.0065	**37.07**	**<0.001**
*V*_block_	**0.0065**	0.0001	0.017	**13.72**	**<0.001**
*V*_R_	0.016	0.013	0.018		
*h^2^*	0.13				
*m^2^*	0.12				
Boldness					
*V*_S_	<0.001	0	0.066	<0.01	>0.999
*V*_M_	0.020	0	0.11	0.19	0.665
*V*_block_	0.029	<0.001	0.22	<0.01	>0.999
*V*_test day_	**0.093**	0	0.12	**5.43**	**0.02**
*V*_R_	2.41	2.14	2.68		
*h^2^*	<0.01				
*m^2^*	0.01				
Total activity					
*V*_S_	<0.01	0	2.99	<0.01	>0.999
*V*_M_	**5.20**	0.88	8.89	**12.36**	**<0.001**
*V*_block_	0	0	2.20	<0.01	>0.999
*V*_test day_	0.20	0	2.01	<0.01	0.990
*V*_R_	58.8	52.1	65.4		
*h^2^*	<0.01				
*m^2^*	0.08				
Activity‐per‐minute					
*V*_S_	<0.01	0	0.063	<0.01	>0.999
*V*_M_	**0.10**	0.0096	0.18	**10.82**	**0.001**
*V*_block_	0	0	0.040	<0.01	>0.999
*V*_test day_	0	0	0.042	<0.01	>0.999
*V*_I (intercept)_	**1.33**	1.17	1.50	**1276.8**	**<0.001**
*V*_I (slope, minute × ID)_	**0.24**	0.45	0.54	**340.08**	**<0.001**
*V*_I (correlation:intercept, ID)_	0.38	0.28	0.47		
*V*_R_	0.71	0.66	0.75		
*h^2^*	<0.01				
*m^2^*	0.05				

Note

Significant components are in bold, based on *p*‐values calculated from LRTs; note that these *p*‐values are conservative and may be up to 2× larger than they should be. *h* ^2^: narrow‐sense heritability; LCI/UCI: 95% lower/upper confidence intervals from 10,000 bootstraps; *m* ^2^: maternal effect; *V*_block_: block variance component; *V*_I_: individual variance component; *V*_M_: mother variance component; *V*_R_: residual variance; *V*_S_: sire variance component; *χ* ^2^: LRT statistic.

**Figure 1:**
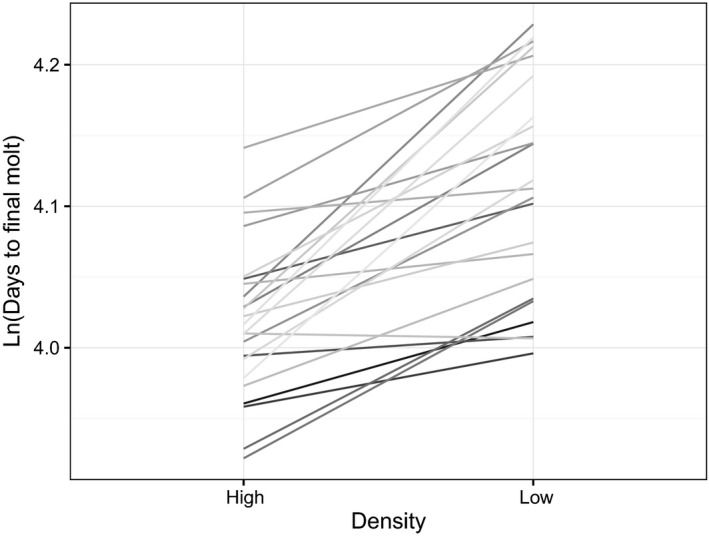
Predicted reaction norms (developmental density × sire) for development time. Each line represents a full‐sib family from a given sire

Development time was the only trait that had a significant density × sire interaction (*χ* ^2^ = 33.72, *p* < 0.001, Figure 1), indicating that paternal families responded differently to rearing density, that is, there was a G × E interaction of density on development time. The correlation between intercept and slope (−0.2, with high density as the reference) indicated that paternal families that had the shortest development times at high density showed a greater increase in development times at low density, compared with paternal families that had longer development times at high density. At low density, *V*_S_ was not significant when the block effect was included (3.7% of *V*_P_, *h^2^* = 0.13), but was significant when the block effect was not included (27.1% of *V*_P_, *h^2^* = 0.60). Similarly, at high density, *V*_S_ was not significant when the block effect was included (3.6% of *V*_P_, *h^2^* = 0.13), but was significant when the block effect was not included (21.5% of *V*_P_, *h^2^* = 0.52). At low density, *V*_M_ was significant whether or not block effect was included (with block: 13.3% of *V*_P_, *m^2^* = 0.12; without block: 13.7% of *V*_P_, *m^2^* = 0.08). Similarly, at high density, *V*_M_ was significant whether or not block effect was included (with block: 19.7% of *V*_P_, *m^2^* = 0.18; without block: 11.1% of *V*_P_, *m^2^* = 0.07).

Boldness did not show significant *V*_S_ in either set of models (<0.1% of *V*_P_ and *h^2^* <0.1 in both cases) nor did it show significant *V*_M_ (with block: 0.8% of *V*_P_, *m^2^* = 0.01; without block: 1.4% of *V*_P_, *m^2^* = 0.06). Total activity did not show significant *V*_S_ in either set of models (<0.1% of *V*_P_ and *h^2^* <0.1 in both cases) but did show significant *V*_M_ in both sets of models (8.1% of *V*_P_, *m^2^* = 0.08 for both). Similarly, activity‐per‐minute did not show significant *V*_S_ in either set of models (<0.1% of *V*_P_ and *h^2^* <0.1 in both cases) but did show significant *V*_M_ in both sets of models (4.8% of *V*_P_, *m^2^* = 0.05 for both). Additionally, individuals differed consistently in how much distance they covered during each minute of the open field and in the slope of their activity over time. A positive correlation between intercept and slope (0.38) indicates that overall activity during the test was linked to a stronger increase in activity with time.

### 3.2 Fixed effects of density and sex

**Figure 2:**
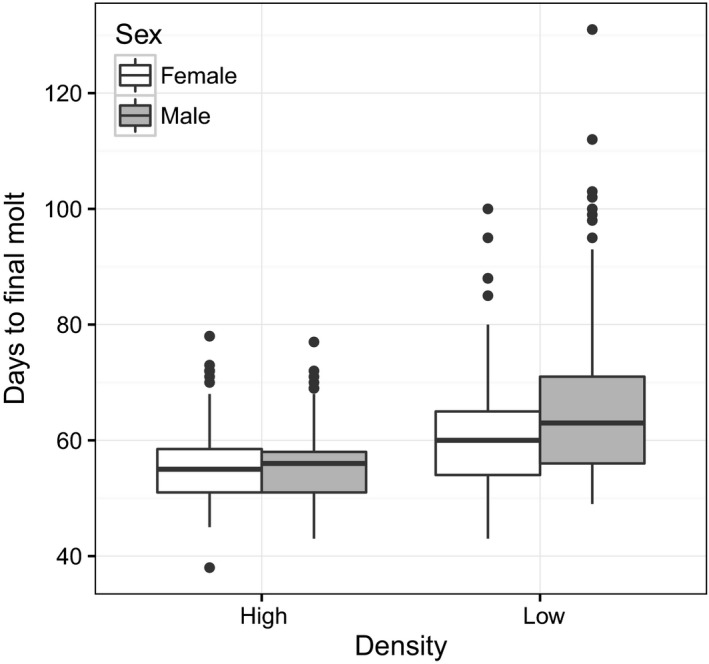
Days from hatching date to eclosion by rearing density treatment and sex

Fixed effects for all models are presented in Table 2. Body mass at eclosion (mean = 0.727 g, *SD* = 0.144, range: 0.313–1.239, *N* = 678) was significantly affected by rearing density and differed by sex. Low‐density crickets were heavier than high‐density crickets, and female crickets were heavier than males. Body mass at sexual maturity (mean = 0.752 g, *SD* = 0.170, range: 0.308–1.424, *N* = 678) showed a similar pattern, with even larger sex differences due to both an average mass gain in females (mean = 0.079 g) and an average mass loss in males (mean = −0.025 g) from 0 to day of behavioral testing. Development time (mean = 59.46 days to eclosion, *SD* = 10.11, range: 38–131, *N* = 670) showed a significant density × sex interaction, such that there was no sex difference at high densities, but male crickets took longer to mature than females at low densities (Figure 2). Individuals of both sexes matured more quickly when reared in high‐density treatments.

**Table 2:** Fixed effects of density and sex on measured traits

Traits/sources	Estimate	LCI	UCI	*p*‐value
Mass, eclosion (g)^a^				
Intercept	**0.77**	0.72	0.81	**<0.001**
Density^b^	**0.05**	0.02	0.06	**<0.001**
Sex^c^	**−0.09**	−0.12	−0.08	**<0.001**
Density^b^ × Sex^c^	−0.02	−0.06	0.01	0.214
Mass, sexual maturity (g)^a^				
Intercept	**0.84**	0.79	0.88	**<0.001**
Density^b^	**0.06**	0.03	0.06	**<0.001**
Sex^c^	**−0.19**	−0.22	−0.19	**<0.001**
Density^b^ × Sex^c^	0.02	−0.06	0.01	0.224
Development time (log [days to eclosion])				
Intercept	**4.02**	3.97	4.08	**<0.001**
Density^b^	**0.10**	0.06	0.13	**<0.001**
Sex^c^	−0.0002	−0.02	0.02	0.989
Density^b^ × Sex^c^	**0.05**	0.02	0.08	**0.004**
Boldness				
Intercept	**2.68**	2.38	2.98	**<0.001**
Density^b^	0.04	−0.31	0.38	0.842
Sex^c^	0.14	−0.19	0.48	0.410
Density^b^ × Sex^c^	0.01	−0.47	0.48	0.974
Days after eclosion^d^	0.16	−0.01	0.32	0.068
Total activity (sqrt [cm])				
Intercept	**25.84**	24.48	27.21	**<0.001**
Density^b^	−0.74	−2.42	0.96	0.394
Sex^c^	**−4.85**	−6.50	−3.20	**<0.001**
Density^b^ × Sex^c^	1.25	−1.12	3.58	0.303
Days after eclosion^d^	−0.38	−1.10	0.33	0.292
Activity‐per‐minute				
Intercept	**4.09**	3.89	4.30	**<0.001**
Density^b^	−0.10	−0.37	0.16	0.448
Sex^c^	**−0.68**	−0.93	−0.42	**<0.001**
Time (scaled [min])	**0.26**	0.18	0.34	**<0.001**
Time^2^	**0.08**	0.04	0.11	**<0.001**
Density^b^ × Sex^c^	0.36	0.003	0.71	0.048
Density^b^ × Time	−0.01	−0.10	0.08	0.813
Sex^c^ × Time	**−0.25**	−0.34	−0.15	**<0.001**
Days after eclosion^d^	−0.07	−0.17	0.03	0.170

Note

Significant effects are in bold; LCI/UCI: 95% lower/upper confidence intervals from 10,000 bootstraps.

a Cricket mass was originally measured to the nearest 0.1 mg.

b The reference density was “high.”

c The reference sex was “female.”

d “Days since eclosion” was centered to mean of 0 and scaled to standard deviation of 1.

Cricket boldness (mean latency to emerge = 265.3 s, *SD* = 379.4, range: 0–1,800, *N* = 679) was not predicted by rearing density or sex, but there was a nonsignificant tendency for more sexually mature animals to be bolder (emerge more quickly). Total activity in the 5‐min open‐field test (mean = 265.3 cm, *SD* = 403.73, range: 52.31–2214.0, *N* = 668) was not affected by density treatment or sexual maturity but did differ by sex. Male crickets covered less distance than females did. Similarly, activity‐per‐minute (mean = 105.10, *SD* = 103.39, range: 0–660.50, *N* = 3,332) was not affected by density treatment or sexual maturity, but did differ by sex, with male crickets covering less distance than females did. The sex difference increased with time, with male crickets becoming less active as time went on. Activity also showed a significant convex quadratic pattern, such that activity increased more steeply as time went on.

## 4 DISCUSSION

We quantified variance components and response to rearing density of behavioral traits in *G. firmus*, a model system for the study of developmental plasticity and life‐history strategies. Behavior (boldness and activity) did not respond to density treatments, nor was it heritable. This contrasted with results for morphology and life history, which responded to treatment density and showed significant genetic components. Animals reared at high densities were generally lighter and took longer to mature. Body mass was moderately heritable, and response of development time to rearing density differed across genotypes. Overall, our results suggest that, in this system, genetic factors play little role in determining variation in the behavioral traits measured and that these behaviors might have relatively limited evolvability and response to selection.

In this system, the behaviors we measured, boldness and activity, were not heritable, although there were significant maternal effects on activity. In this specific case, the “phenotypic gambit” of assuming behavioral variation reflects genotypic variation would not be well supported. These results suggest that activity and boldness might have limited potential response to selection in this system but are possibly being affected by other unmeasured environmental factors, as there were significant effects of testing day (on boldness) and mother (on activity). Other studies have commonly documented some degree of heritability in a wide variety of behaviors, often of a similar magnitude to life‐history traits (Stirling et al., 2002). However, a study similar to ours in *G. integer* also found only weak to marginal heritability for boldness measures (Niemelä et al., 2013). In our system, behavioral traits were highly variable within as well as among families and between treatments. Perhaps similar to the high degree of within‐clutch variation seen in other traits (e.g., wing polymorphisms, hatching rate), high within‐family behavioral variation might be an adaptation to the unstable natural habitats of this species. Female *G. firmus* are also known to exhibit behavioral compensation (i.e., plasticity) in oviposition (Réale & Roff, 2002), indicating a large degree of behavioral flexibility in this other context. We measured behavioral traits on a short timescale and in the broad contexts of activity and response to potential risk. There has been much discussion on the most relevant approaches for studying behavioral traits at the individual level (Dingemanse & Dochtermann, 2013; Réale et al., 2007; Stamps & Groothuis, 2010a, 2010b), and more studies might be necessary to identify particular behavioral traits or contexts most relevant to density‐dependent plasticity. For example, Kasumovic and Brooks (2011) suggest that adaptively cued social plasticity (phenotypic plasticity in response to social cues during development in anticipation of adult conditions) should be most relevant to mating contexts. An interesting extension to the current study would be to further examine the ability of various traits to respond to selection (their evolvability) using mean‐standardized measures, which have been suggested as more appropriate for comparing genetic variability (Houle, 1992).

Over the short timescale of the behavioral test, we did find large among‐individual differences in activity‐per‐minute, as well as change in activity over time. While we did not measure individual repeatability of behavioral traits over multiple tests, a positive correlation between activity in the first minute and total activity in the 5‐min. open field (*r* = 0.59, results not shown) in combination with the individual differences in activity‐per‐minute suggests that individuals differed relatively consistently on this short timescale. For future tests, activity in the first minute of the open field might be an adequate behavioral measure and has the advantages of being easier to collect and likely to be less influenced by factors occurring later in the test. In general, more analyses of temporal activity patterns in open field and other behavioral tests will facilitate more detailed understanding of individual and group variation, allow better comparison of results from different studies, and test for potential biases of different test lengths (Montiglio, Garant, Thomas, & Réale, 2010).

Counter to expectations, we did not find significant fixed effects of density treatments on the expression of behavioral traits. Instead, significant effects of test day (on boldness) and of mother (on activity) suggest other unmeasured environmental influences on behavior. This result differs from the common observation of behavioral responses to rearing density (Dingle, 2014; Applebaum & Heifetz, 1999; Lihoreau, Brepson, & Rivault, 2009) or social cues (DiRienzo, Pruitt, & Hedrick, 2012) across insect systems. However, Niemelä, Vainikka, Lahdenperä et al. (2012b) also found little effect of rearing density on boldness or aggressiveness in adults in *G. integer*. Other studies have suggested that developmental experience can influence more subtle aspects of behavioral expression, without affecting the mean. DiRienzo, Niemelä, Skog, Vainikka, and Kortet (2015) found that early exposure to pathogens did not affect mean adult boldness but rather the repeatability of this behavior in *G. integer*, and Royauté and Dochtermann (2017) found that developmental diet affected behavioral variances but not means in the house cricket, *Acheta domesticus*. However, we did not find an effect of density on variance components explaining behavioral traits. We did find a sex difference in total activity in the open field, with male crickets traveling significantly shorter distances overall, although there was no sex difference for the first minute of activity. This indicates that, while both sexes were equally active at the beginning of the behavioral trial, male crickets generally become less active than females as the trial went on. We observed from videos that male crickets would sometimes settle into a corner facing out into the arena, as if defending the corner, so a possible hypothesis is that territorial behavior affected activity in males, but this remains to be tested. Hence, the behaviors measured here could be influenced by factors such as reproductive state or other individual differences.

In contrast with results for behavior, our results for body mass at eclosion, body mass at sexual maturity, and development time generally aligned with expectations based on life‐history theory and are consistent with previous findings in this and other systems (Stearns, 1992; Roff, 1993). Animals reared at higher density matured more quickly and at a lower body mass on average than animals reared at low densities, a widely seen pattern in insects (Applebaum & Heifetz, 1999; Niemelä, Vainikka, Lahdenperä et al., 2012b). The difference in development time between densities was more pronounced for male crickets. Males might be more responsive to social cues because competition for mating opportunities is likely to be higher in males (Andersson, 1994). Differences in population density are expected to change levels of competition, and many studies have shown that life‐history traits and behavior can be influenced by population density in many species (Guo, Mueller, & Ayala, 1991; Mueller, Guo, & Ayala, 1991). We also observed that female crickets were heavier than males and gained mass posteclosion, while males tended to lose mass posteclosion, a pattern consistent with other studies in crickets showing sex differences in mass gain posteclosion. Fecundity is highly dependent on body mass and size in female crickets; thus, reproductive benefits of body size differ between the sexes, and this has been an explanation for why sexual dimorphism has evolved in crickets (Hunt et al., 2004; Judge, Ting, & Gwynne, 2008; Kelly & Tawes, 2013).

Adult body mass and development time showed significant sire components and were heritable, with estimates of heritability (mass at eclosion *h* ^2^ = 0.45–0.50; development time *h* ^2^ = 0.52–0.60; in models not including block effect) similar to those previously reported for *G. firmus* (Roff, 1998), other *Gryllus* species (Niemelä et al., 2013), and across taxa for life‐history traits (Mousseau & Roff, 1987; Stirling et al., 2002). Interestingly, development time further showed a significant density by sire interaction, indicating that genotypes responded differently to rearing density. For this trait, these differences in plasticity could also be the target of selection (West‐Eberhard, 1989). The negative correlation between intercept and slope indicated that genotypes with the shortest development times at high density showed a greater increase in development times at low density, compared with genotypes with longer development times at high density. There were also significant differences among mating blocks in both measures of body mass and development time, revealing some unmeasured temporal variation in the parental generation that affected these traits.

Several aspects of our study design warrant further discussion of their potential effects on interpretation of results. First, as we did not have repeated measures of behavioral traits, we cannot rule out the possibility that the lack of heritability and density effects were due to the behavioral measures failing to represent consistent interindividual variation. Given that previous work detected repeatability of boldness in *Gryllus* with much smaller sample sizes and that substantial work has shown density effects on activity in many species, we believe our results are unlikely to be entirely due to this issue. However, we acknowledge this issue limits our interpretation of results. Additionally, because experimental families were created over the course of a few months within one generation, some parental components cannot be untangled from temporal components. For example, we could not remove the effect of earlier developing individuals being used as parents in earlier blocks, that is, sires and dams were assigned to blocks as they matured, rather than randomly. In our study, heritability decreased, and *V*_S_ was not significant for development time when accounting for block. Including or removing block from the model mostly affected variance among sires for life‐history traits but did not have any effects on behavioral traits or on among‐dam variance. This result suggests that variance among blocks was partly caused by genetic variance rather than the opposite and that thus the model with block might be underestimating sire effects. Another important consequence of the study design is that, for practical reasons and to reduce variation from these sources, experimental animals were limited to relatively fast‐hatching and fast‐maturing individuals in the experiment. Hence, we have less insight on later‐maturing animals and cannot generalize to animals that would have emerged from diapause eggs. Consequently, this study likely reflects a much narrower range of trait variation than is present in the system.

Developmental plasticity is just one way that organisms can respond to environmental uncertainty, and our results for behavioral traits might be more consistent with a bet‐hedging strategy (Donaldson‐Matasci, Lachmann, & Bergstrom, 2008; Philippi & Seger, 1989), where a variety of behavioral phenotypes exist within families, thus, increasing the chances that some offspring will be matched to a range of environments. This evolutionary strategy might be more adaptive in environments that are unstable and, therefore, unlikely to provide reliable cues for matching phenotypes to anticipated future conditions (Pigliucci, 2005; Shuster & Wade, 2003; West‐Eberhard, 2003). *Gryllus firmus* inhabits highly variable sandy habitats, and, within the same clutch, females can lay a mixture of fast‐developing and diapause eggs (Walker, 1980) as well as a mixture of long‐ and short‐wing morphs (Roff, 1990). Additionally, given the prominent wing dimorphism that allows some individuals to disperse away from current conditions, behavioral traits may not be selected to respond to rearing conditions because many individuals have the opportunity to disperse and encounter adult conditions different from developmental conditions. It is also possible that individuals might show different strategies, among or within families, that are not apparent from family phenotypes, and the correlation structure among traits is the subject of ongoing research. The expression of behavioral traits, like life‐history traits, will be influenced by many interacting and sometimes competing genetic and environmental factors, and much remains to be studied about their joint effects on behavioral variation.

## CONFLICT OF INTEREST

None declared.

## AUTHOR CONTRIBUTIONS

All authors contributed to the conceptualization and design of the study. TWW conducted the experiment, collected and analyzed data, and wrote the initial manuscript with input from DR and CDK. All authors contributed to the critical evaluation of results and revisions of the manuscript.

## Data Availability

Data are available at Dryad (https://doi.org/10.5061/dryad.gg7rt25). Data can also be requested directly from the authors.
