# Supplementary material for: Developmental and genetic effects on behavioral and life‐history traits in a field cricket
Source: Ecol Evol. 2019 Feb 23;9(6):3434–45. doi: 10.1002/ece3.4975 (PMC6434558; doi:10.1002/ece3.4975)
Supplement: Supplementary file 1 [file ECE3-9-3434-s001.docx]

Supplementary Information

Table S1. Variance components from models without Block effect

| Variance Components | Predictor | | LCI | UCI | | χ^2^ | | p-value | |
| --- | --- | --- | --- | --- | --- | --- | --- | --- | --- |
| Mass, eclosion |  |  |  |  |  | |  | |  |
| *V_S_* |  | **3788** | 1557 | 6642 | **29.04** | | **< 0.001** | |  |
| *V_M_* |  | 0 | 0 | 665 | < 0.01 | | > 0.999 | |  |
| *V_R_* |  | 14440 | 12832 | 15987 |  | |  | |  |
| *h^2^* |  | 0.51 |  |  |  | |  | |  |
| *m^2^* |  | < 0.01 |  |  |  | |  | |  |
| Mass, sexual maturity |  |  |  |  |  | |  | |  |
| *V_S_* |  | **3071** | 1078 | 5729 | **15.35** | | **< 0.001** | |  |
| *V_M_* |  | **715** | < 0.01 | 1766 | **4.05** | | **0.044** | |  |
| *V_R_* |  | 14421 | 12839 | 16060 |  | |  | |  |
| *h^2^* |  | 0.45 |  |  |  | |  | |  |
| *m^2^* |  | 0.03 |  |  |  | |  | |  |
| Development time |  |  |  |  |  | |  | |  |
| *Low density* |  |  |  |  |  | |  | |  |
| *V_S_* |  | **0.0072** | 0.0020 | 0.014 | **14.71** | | **< 0.001** | |  |
| *V_M_* |  | **0.0036** | < 0.001 | 0.0070 | **13.64** | | **< 0.001** | |  |
| *V_R_* |  | 0.016 | 0.013 | 0.018 |  | |  | |  |
| *h^2^* |  | 0.60 |  |  |  | |  | |  |
| *m^2^* |  | 0.08 |  |  |  | |  | |  |
| *High density* |  |  |  |  |  | |  | |  |
| *V_S_* |  | **0.0050** | 0.0016 | 0.0045 | **13.50** | | **< 0.001** | |  |
| *V_M_* |  | **0.0026** | 0.0011 | 0.0095 | **28.08** | | **< 0.001** | |  |
| *V_R_* |  | 0.0062 | 0.0062 | 0.0073 |  | |  | |  |
| *h^2^* |  | 0.52 |  |  |  | |  | |  |
| *m^2^* |  | 0.07 |  |  |  | |  | |  |
| Boldness |  |  |  |  |  | |  | |  |
| *V_S_* |  | < 0.01 | 0 | 0.076 | < 0.01 | | > 0.999 | |  |
| *V_M_* |  | 0.036 | 0.00 | 0.13 | 0.55 | | 0.457 | |  |
| *V_test day_* |  | **0.10** | < 0.01 | 0.24 | **5.93** | | **0.015** | |  |
| *V_R_* |  | 2.41 | 2.15 | 2.69 |  | |  | |  |
| *h^2^* |  | < 0.01 |  |  |  | |  | |  |
| *m^2^* |  | 0.01 |  |  |  | |  | |  |
| Total activity |  |  |  |  |  | |  | |  |
| *V_S_* |  | 0 | 0 | 3.17 | < 0.01 | | > 0.999 | |  |
| *V_M_* |  | **5.20** | 0.94 | 9.04 | **12.36** | | **< 0.001** | |  |
| *V_test day_* |  | 0.20 | 0 | 2.25 | < 0.01 | | > 0.990 | |  |
| *V_R_* |  | 58.84 | 52.08 | 65.35 |  | |  | |  |
| *h^2^* |  | < 0.01 |  |  |  | |  | |  |
| *m^2^* |  | 0.08 |  |  |  | |  | |  |
| Activity-per-minute |  |  |  |  |  | |  | |  |
| *V_S_* |  | 0 | 0 | 0.064 | < 0.01 | | > 0.999 | |  |
| *V_M_* |  | **0.10** | 0.0097 | 0.18 | **10.82** | | **0.001** | |  |
| *V_test day_* |  | 0 | 0 | 0.043 | < 0.01 | | > 0.999 | |  |
| *V_I (intercept)_* |  | **1.33** | 1.17 | 1.50 | **1276.8** | | **< 0.001** | |  |
| *V_I (slope, minute x ID)_* |  | **0.24** | 0.20 | 0.29 | **340.08** | | **< 0.001** | |  |
| *V_I (correlation:intercept, ID)_* |  | 0.38 | 0.28 | 0.47 |  | |  | |  |
| *V_R_* |  | 0.71 | 0.66 | 0.75 |  | |  | |  |
| *h^2^* |  | < 0.01 |  |  |  | |  | |  |
| *m^2^* |  | 0.05 |  |  |  | |  | |  |

Significant components are in bold, based on p-values calculated from LRTs; note that these p-values are conservative and may be up to 2x larger than they should be. *V_S_* = sire variance component; *V_M_* = mother variance component; *V_R_* = residual variance; *V_I_* = individual variance component; χ^2^ = LRT statistic; *h*^2^ = narrow-sense heritability; *m*^2^ = maternal effect; LCI/UCI = lower/upper confidence intervals from 10 000 bootstraps.

Table S2. Treatment-specific variance components from models with Block effect

|  | High Density | | | | | Low Density | | | | |
| --- | --- | --- | --- | --- | --- | --- | --- | --- | --- | --- |
| Variance Components | Predictor | 95% LCI | 95% UCI | χ^2^ | p-value | Predictor | 95% LCI | 95% UCI | χ^2^ | p-value |
| Mass, eclosion |  |  |  |  |  |  |  |  |  |  |
| *V_S_* | 677 | 0 | 2012 | 1.45 | 0.228 | **1498** | < 0.01 | **3478** | **6.80** | **0.009** |
| *V_M_* | 247 | 0 | 1524 | 0.17 | 0.677 | < 0.01 | 0 | 1416 | < 0.01 | > 0.999 |
| *V_block_* | 1979 | 0 | 5675 | < 0.01 | > 0.999 | 2659 | 0 | 7787 | < 0.01 | > 0.999 |
| *V_R_* | 13211 | 11072 | 15296 |  |  | 15500 | 12974 | 17903 |  |  |
| *h^2^* | 0.15 |  |  |  |  | 0.25 |  |  |  |  |
| *m^2^* | 0.01 |  |  |  |  | < 0.01 |  |  |  |  |
| Mass, sexual maturity |  |  |  |  |  |  |  |  |  |  |
| *V_S_* | 0 | 0 | 1426 | < 0.01 | > 0.999 | 1007 | 0 | 2598 | 3.61 | 0.057 |
| *V_M_* | **1916** | 188 | 3523 | **9.19** | **0.002** | 0 | 0 | 1390 | < 0.01 | > 0.999 |
| *V_block_* | 2195 | < 0.01 | 6119 | < 0.01 | > 0.999 | **2950** | < 0.01 | **8300** | **12.25** | **< 0.001** |
| *V_R_* | 12407 | 10416 | 14511 |  |  | 15697 | 13129 | 18030 |  |  |
| *h^2^* | < 0.01 |  |  |  |  | 0.18 |  |  |  |  |
| *m^2^* | 0.12 |  |  |  |  | < 0.01 |  |  |  |  |
| Development time* |  |  |  |  |  |  |  |  |  |  |
| *V_S_* | 0.0005 | 0 | 0.0022 | 0.35 | 0.556 | 0.0010 | 0 | 0.0040 | 0.08 | 0.783 |
| *V_M_* | **0.0026** | 0.0011 | 0.0043 | **27.25** | **< 0.001** | **0.0036** | 0.0010 | 0.0065 | **37.07** | **< 0.001** |
| *V_block_* | **0.0039** | 0.0002 | 0.011 | **11.58** | **< 0.001** | **0.0065** | 0.0001 | 0.017 | **13.72** | **< 0.001** |
| *V_R_* | 0.0062 | 0.0052 | 0.0073 |  |  | 0.016 | 0.013 | 0.018 |  |  |
| *h^2^* | 0.13 |  |  |  |  | 0.13 |  |  |  |  |
| *m^2^* | 0.18 |  |  |  |  | 0.12 |  |  |  |  |
| Boldness |  |  |  |  |  |  |  |  |  |  |
| *V_S_* | 0 | 0 | 0.11 | < 0.01 | > 0.999 | 0 | 0 | 0.16 | < 0.01 | > 0.999 |
| *V_M_* | 0 | 0 | 0.17 | < 0.01 | > 0.999 | < 0.001 | 0 | 0.18 | < 0.01 | > 0.999 |
| *V_block_* | 0.027 | 0 | 0.16 | < 0.01 | > 0.999 | 0.047 | 0 | 0.21 | < 0.01 | > 0.999 |
| *V_test day_* | 0.13 | 0 | 0.35 | < 0.01 | > 0.999 | 0.041 | 0 | 0.22 | < 0.01 | > 0.999 |
| *V_R_* | 2.36 | 1.95 | 2.71 |  |  | 2.51 | 2.06 | 2.86 |  |  |
| *h^2^* | < 0.01 |  |  |  |  | < 0.01 |  |  |  |  |
| *m^2^* | < 0.01 |  |  |  |  | < 0.01 |  |  |  |  |
| Total activity |  |  |  |  |  |  |  |  |  |  |
| *V_S_* | 0 | 0 | 5.89 | < 0.01 | > 0.999 | 0.56 | 0 | 4.36 | 0.03 | 0.872 |
| *V_M_* | **10.28** | 1.45 | 17.90 | **10.74** | **0.001** | 3.12 | 0 | 8.34 | 1.53 | 0.22 |
| *V_block_* | 0 | 0 | 3.99 | < 0.01 | > 0.999 | < 0.01 | 0 | 2.76 | < 0.01 | > 0.999 |
| *V_test day_* | 0.66 | 0 | 5.00 | < 0.01 | > 0.999 | 0.95 | 0 | 5.05 | < 0.01 | > 0.999 |
| *V_R_* | 59.65 | 49.40 | 69.80 |  |  | 53.57 | 44.25 | 62.44 |  |  |
| *h^2^* | < 0.01 |  |  |  |  | 0.04 |  |  |  |  |
| *m^2^* | 0.15 |  |  |  |  | 0.05 |  |  |  |  |
| Activity-per-minute |  |  |  |  |  |  |  |  |  |  |
| *V_S_* | 0 | 0 | 0.09 | < 0.01 | > 0.999 | 0 | 0 | 0.30 | < 0.01 | > 0.999 |
| *V_M_* | **0.19** | 0.005 | 0.34 | **8.30** | **0.004** | 0.11 | 0 | 0.23 | 3.35 | 0.067 |
| *V_block_* | 0 | 0 | 0.09 | < 0.01 | > 0.999 | 0 | 0 | 0.06 | < 0.01 | > 0.999 |
| *V_test day_* | 0.03 | 0 | 0.14 | < 0.01 | > 0.999 | 0 | 0 | 0.08 | < 0.01 | > 0.999 |
| *V_I (intercept)_* | **1.30** | 1.07 | 1.56 | **589.19** | **< 0.001** | **1.26** | 1.02 | 1.49 | **567.65** | **< 0.001** |
| *V_I (slope, minute x ID)_* | **0.27** | 0.17 | 0.29 | **203.77** | **< 0.001** | **0.21** | 0.13 | 0.23 | **138.52** | **< 0.001** |
| *V_I (correlation:intercept, ID)_* | 0.39 | 0.26 | 0.51 |  |  | 0.40 | 0.27 | 0.54 |  |  |
| *V_R_* | 0.68 | 0.62 | 0.74 |  |  | 0.73 | 0.67 | 0.80 |  |  |
| *h^2^* | < 0.01 |  |  |  |  | < 0.01 |  |  |  |  |
| *m^2^* | 0.08 |  |  |  |  | 0.05 |  |  |  |  |

Significant components are in bold, based on p-values calculated from LRTs; note that these p-values are conservative and may be up to 2x larger than they should be. *V_S_* = sire variance component; *V_M_* = mother variance component; *V_block_* = block variance component; *V_R_* = residual variance; *V_I_* = individual variance component; χ^2^ = LRT statistic; *h*^2^ = narrow-sense heritability; *m*^2^ = maternal effect; LCI/UCI = lower/upper confidence intervals from 10 000 bootstraps. *For Development time, variance components are the same as in Table 1.
